# A cost of illness analysis of hepatocellular carcinoma for the Greek healthcare setting 

**Published:** 2020

**Authors:** Kostas Athanasakis, Fani Pliarchopoulou, Vasiliki Naoum, Christos Psarrakis, Nikolaos Tziolos, Theodoros Marantos, Christina Damoulari, Athina Chounta

**Affiliations:** 1 *Department of Public Health Policy, School of Public Health, University of West Attica, Athens, Greece*; 2 *4th Department of Internal Medicine, University General Hospital "ATTIKON", National and Kapodistrian University of Athens, Haidari, Athens, Greece*; 3 *Institute for Health Economics, Athens, Greece*

**Keywords:** Cost-of-illness, Hepatocellular cancer, Economic evaluation

## Abstract

**Aim:**

To estimate the cost per patient for hepatocellular carcinoma in Greece, a setting that is currently facing financial constraints.

**Background:**

Hepatocellular carcinoma patient management strategies are associated with significant costs. Despite this, patient level data on healthcare resource use and cost-of-illness analyses of hepatocellular carcinoma remain rather scarce in the international literature.

**Methods:**

123 patients diagnosed with hepatocellular carcinoma and followed in a specialised clinic of a tertiary hospital in Greece formed the basis of the analysis. Detailed resource use data were derived from the medical records of each patient. Data were recorded from the first encounter of the patient with the facility until a fatal endpoint or until the last day of follow up. Patients that were lost to follow-up were excluded from the analysis. Calculations follow a third-party payer perspective, according to official prices and tariffs.

**Results:**

The average cost per patient was estimated at 12,119.1 Euros (SD: 14,670.3) (21,375.1 PPP USD) for the average follow-up period and 10,241.5 Euros (18,063.5 PPP USD) per year. Median costs per month of follow-up according to underlying disease were 1,218.1, 1,376.8, 1,521.3 and 686.9 Euros (2,148.4, 2,428.3, 2,683.2 and 1,211.5 PPP USD) for patients with alcoholic steatohepatitis, hepatitis B, hepatitis C and non-alcoholic fatty liver disease, respectively.

**Conclusion:**

Hepatocellular carcinoma represents a heavy toll, both from the clinical as well as from the economic perspective, especially for a setting in “dire straits”. Interventions towards reducing the incidence and, subsequently, the cost of HCC are imperative.

## Introduction

 The burden of Hepatocellular carcinoma (HCC) in terms of incidence and mortality in Europe has been significantly rising during the last decades. Currently, HCC is the 12^th^ most frequent cancer, in terms of incidence among cancer cases in Europe and the fifth contributor, in terms of mortality (1).

HCC patient management strategies are complex, require the use of multiple and technologically advanced healthcare resources and, thus, are associated with significant costs. Nevertheless, accurate estimates of HCC costs remain sparse, (2) especially in the European setting (3). This lack of up-to-date, context specific and detailed estimates creates an evidence gap, which is often overlooked. HCC cost inputs are very frequently utilised as input data in cost-effectiveness analyses for hepatitis medications – especially in the rapidly rising health economics literature of chronic hepatitis C (HCV)-, as bases of the estimates of total burden of disease for liver disorders as well as inputs for population interventions, such as surveillance or screening programs, (4) that aim to combat the increasing burden of hepatitis. The lack of actual estimates leads to the use of approximations that are lacking in terms of accuracy and, thus, limit the level of evidence provided by the models that rely on them as inputs.

In light of this gap and with an aim to contribute towards the availability of country specific estimates on the cost of HCC, especially in resource limited settings, such as Greece in the post-economic crisis period, the objective of this analysis was to estimate and provide the first estimate on the costs associated with the management of HCC patients in Greece – and one of the few detailed calculations on HCC costs existing in the literature. 

## Methods

The methodology of our study adheres to the standards of the micro-costing approach in cost-of-illness analyses (5). The primary data for the analysis are sourced from a retrospective review of the medical records of 123 patients diagnosed with HCC and monitored in the Hepatology Unit of a tertiary hospital. To avoid selection bias, investigators identified potentially eligible patients from consecutive visits in the clinic visit register. Starting with the patient with the most recent visit, patients with consecutive visits (backwards in time) were screened. Inclusion criteria were a) age >18 years b) diagnosis of HCC c) having complete data (i.e. being on active follow-up or having deceased at a documented date). Patients that were lost to follow-up were excluded from the analyses. For the purposes of the study, an approval from the hospital’s Bioethics committee was obtained (Nr6/07.04.15).

The data were extracted from the patient’s medical file starting from the date of diagnosis until the last date of follow-up. The collected data comprised patient demographic and clinical characteristics, underlying disease (hepatitis B, hepatitis C, alcoholic steatohepatitis (ASH), non-alcoholic fatty liver disease (NAFLD) or comorbidities), and health-resource utilisation (medications, laboratory tests, biopsies, imaging tests, physician visits, and hospitalisation).

The annual direct medical cost was calculated taking into account the resources used and the unitary costs from the third party payer’s perspective (social insurance). Cost of medications was taken from the official drug Price Bulletin. The costs for inpatient hospital stays were calculated according to the Greek DRG system. The cost of biopsies, laboratory tests as well as the cost of visits to specialists are based on official tariffs. All unit costs and cost calculations correspond to year 2018 values and are presented in Euros and 2018 USD Purchasing Power Parities (PPP). Conversion to PPP was based on relevant data extracted from the OECD database (6). 

## Results

Patients in the sample were 77.2% male with an average age of 73.1 (SD: 11.5) years and an average follow-up of 14.2 months (SD: 25.3). Cirrhosis was present in 83.7% of the sample participants. Considering underlying diseases, 36.6% of the sample had been diagnosed with hepatitis B, 17.1% with hepatitis C, 22.0% with ASH and 7.3% with NAFLD. Table 1 provides the average resource use (for the most commonly used healthcare resources) and the average cost per patient during the follow-up period in the study sample. 

Overall, the average cost per patient for the 14.2-month follow-up period was 12,119.1 Euros (21,375.1 PPP USD) with a significant variance (SD: 14,670.3). The main driver of the average cost per patient was the cost of hospitalisation (6,716.2 Euros or 11,845.7 PPP USD), followed by the cost of interventional procedures (3,429.7 Euros or 6,049.2 PPP USD) and the cost of medications (1,292.4 Euros or 2,279.5 PPP USD). On an annualized basis, the average cost was 10,241.5 Euros (18,063.5 PPP USD) for the entire sample.

On an individual patient basis, median cost per month of follow up was higher for patients with cirrhosis compared to non-cirrhotic individuals (1,332.1 Euros (2,349.5 PPP USD) vs. 1,199.7 Euros (2,116.0 PPP USD), respectively). Median costs per month of follow-up according to underlying disease were 1,218.1 Euros (2,148.4 PPP USD), 1,376.8 Euros (2,428.3 PPP USD), 1,521.3 Euros (2,683.2 PPP USD) and 686.9 Euros (1,211.5 PPP USD) for patients with ASH, hepatitis B, hepatitis C and NAFLD, respectively.

**Table 1:** Health-resource use and costs for the study sample during the follow-up period

Resource	Mean resource use (standard deviation) or %	
Physician visits	2.9 (0.5)	
Hospitalisation days	19.9 (19.5)	
Biochemistry panels	4.1 (4.7)	
Ultrasounds	1.35 (0.9)	
Immunology assays	1.1 (0.5)	
MRI examinations	1.7 (1.8)	
CT examinations	1.4 (1.3)	
Lipid profiling tests	2.6 (3.4)	
Thyroid function tests	1.6 (1.5)	
Patients that underwent surgical interventions	41.4%	
Costs	Mean per patient costs (median, standard deviation, range). Values in Euros	Mean per patient costs. Values in PPP (USD)
Physician visits	29.1 (10.0, 43.6, 260.0)	51.3
Hospitalisations	6,716.2 (4,025.3, 8,498.9, 55,460.2)	11,845.7
Labwork	651.5 (358.3, 930.7, 5,547.2)	1,149.1
Cost of medications	1,292.4 (0.0, 7,368.7, 78,349.0)	2,279.5
Cost of interventional procedures	3,429.7 (0.0, 5,581.1, 21,710.6)	6,049.2
Total average cost	12,119.1 (8,689.4, 14,670.3, 120,649.8)	21,375.1

MRI: Magnetic Resonance Imaging, CT: Computed Tomography, PPP: Purchasing Power Parities

## Discussion

The results of the present study demonstrate the healthcare resource use and the cost per patient with HCC in Greece. HCC entails a heavy economic burden for the healthcare system that in our study is mainly attributable to the need for secondary/tertiary care. 

Health care of HCC patients entails higher costs compared to non-HCC patients (7). According to the results of our analysis, the total average cost of HCC was estimated at 12,119.1 Euros (21,375.1 PPP USD). Indicatively, when taking into account the average annual gross earnings in the country (28,241.0 Euros (8) or 49,810.2 PPP USD) the total costs due to HCC for a patient in their active years present a multifold increase. Our results suggested that the main driver of the total average cost of HCC was the cost of hospitalisation. Findings from the international literature have also suggested that the cost of hospital care is the main driver of the total cost of the disease. (2, 7, 9, 10)

The cost of the disease may vary according to the disease stage, and possible comorbidities and underlying diseases. According to the international literature, HCC cost for patients with advanced cirrhosis is higher while for patients with mild/moderate cirrhosis the relevant cost is lower (10). The present analysis did not assess the cost of the disease per cirrhosis severity, but estimated the cost per underlying disease. Specifically, the median HCC cost per month of follow-up per underlying disease was estimated to be higher for patients with hepatitis C ***********please CHECK the previous sentence, it was ambiguous*************(1,521.3 Euros or 2,683.2 PPP USD), followed by hepatitis B (1,376.8 Euros or 2,428.3 PPP USD), ASH (1,218.1 Euros or 2,148.4 PPP USD) and NAFLD (686.9 Euros or 1,211.5 PPP USD).

As in every study, the present study has some strengths and limitations. The study is valuable to the literature with respect to the cost of HCC because it is based on real patient data. Even though cost estimations concern solely a tertiary unit, the results of the analysis are considered important since no relevant study on the cost of HCC has been conducted before in Greece. Moreover, the tertiary unit of choice is a referral centre for HCC which monitors not only patients with advanced disease, but also patients with less progressed disease and thus, the cost estimations may be considered indicative of the average HCC cost per patient for the third-party payer. 

Additionally, the estimate of total average annual cost for patients with HCC in this study refers only to the burden on the system (the third-party payer) and does not include indirect costs, i.e. cost due to forgone productivity or the need for informal care. According to findings in the literature, the productivity losses due to HCC account for 10.8% of the total cost of the disease, (9) while informal caregiving in cancer represents 18-33% of the total cost of cancer. (11). 

Moreover, the present estimates refer to the Greek setting and, as with the case of all economic evaluations, are not directly transferable across countries. However, due to the scarcity of micro-costing HCC cost estimates in the literature, the results can serve as a basis on HCC cost inputs in economically comparable or neighbouring countries, following an adjustment for country-specific price levels. 

In light of the morbidity and economic burden associated with the disease, interventions to combat HCC are necessary. However, within the limited budgets that health systems internationally are required to operate, the efficiency (cost-effectiveness) of such interventions must be demonstrated – and in doing so, data of the kind that is presented in this study is necessary.

## Conflict of interests

The authors declare that they have no conflict of interest.
